# Chemical Protein Unfolding – A Simple Cooperative Model

**DOI:** 10.1021/acs.jpcb.3c03558

**Published:** 2023-09-22

**Authors:** Joachim Seelig, Anna Seelig

**Affiliations:** Biozentrum, University of Basel, Spitalstrasse 41, CH-4056 Basel, Switzerland

## Abstract

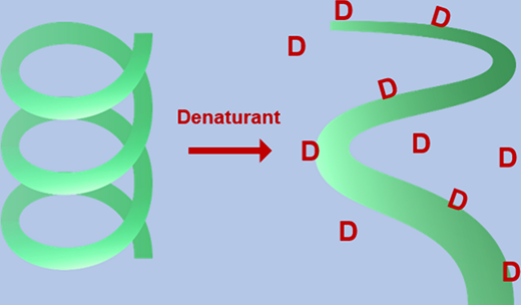

Chemical unfolding with guanidineHCl or urea is a common method to study the conformational stability of proteins. The analysis of unfolding isotherms is usually performed with an empirical linear extrapolation method (LEM). A large positive free energy is assigned to the native protein, which is usually considered to be a minimum of the free energy. The method thus contradicts common expectations. Here, we present a multistate cooperative model that addresses specifically the binding of the denaturant to the protein and the cooperativity of the protein unfolding equilibrium. The model is based on a molecular statistical–mechanical partition function of the ensemble, but simple solutions for the calculation of the binding isotherm and the associated free energy are presented. The model is applied to 23 published unfolding isotherms of small and large proteins. For a given denaturant, the binding constant depends on temperature and pH but shows little protein specificity. Chemical unfolding is less cooperative than thermal unfolding. The cooperativity parameter σ is at least 2 orders of magnitude larger than that of thermal unfolding. The multistate cooperative model predicts zero free energy for the native protein, which becomes strongly negative beyond the midpoint concentration of unfolding. The free energy to unfold a cooperative unit corresponds exactly to the diffusive energy of the denaturant concentration gradient necessary for unfolding. The temperature dependence of unfolding isotherms yields the denaturant-induced unfolding entropy and, in turn, the unfolding enthalpy. The multistate cooperative model provides molecular insight and is as simple to apply as the LEM but avoids the conceptual difficulties of the latter.

## Introduction

Chemical denaturation is a process by which the protein conformation is unfolded via addition of denaturants such as guanidineHCl, urea, or SDS (sodium dodecyl sulfate). Chemical denaturation is a common method for determining a protein’s conformational stability, relative to its functional properties.^1,2^ Identifying the conditions that maximize the structural stability of a protein is crucial during the development of biologics for therapeutic treatments. Several complementary techniques should be applied to provide a systematic analysis of protein stability.^3^

Chemical unfolding of proteins is analyzed almost exclusively with a 2-state model, the linear extrapolation method (LEM).^4^ The LEM is an empirical approximation, and its conceptual difficulties have been realized since its initial proposal.^4−6^ In particular, the LEM assigns a large positive Gibbs free energy, Δ*G*_0_^H_2_O^to the native protein. However, “the general understanding in the protein folding field has been that proteins fold into their native conformations driven by the decrease in Gibbs free energy (negative Δ*G*).”^7^ This thermodynamic hypothesis has become the default physical description of protein folding. In this view, the native state is the most stable one, that is, the global *G* minimum, not a maximum.

The 2-state model is a noncooperative model. It has no energy parameter for the interaction between neighboring amino acid residues. All amino acid residues unfold simultaneously (all-or-none model).^8^ However, the conformational change of all amino acid residues at the same time is physically unrealistic. Instead, “peptides that form helices in solution do not show a simple two-state equilibrium between a fully folded and fully unfolded structure. Instead, they form a complex mixture of all helix, all coil, or, most frequently, central helices with frayed coil ends”.^9^ A sequential cooperative unfolding of protein domains is, therefore, a physically more realistic alternative.

We have recently proposed a semiempirical model that describes a cooperative protein unfolding.^10^ The model assumes explicitly the binding of the denaturant D to the protein with the binding constant *K*_D_ and the cooperative unfolding of the protein with the cooperativity parameter σ.^10^ Here, we provide a modification of this model, based on a statistical-mechanical partition function leading to simple expressions for the chemical unfolding isotherm and the associated free energy.

Published chemical unfolding isotherms obtained with spectroscopic techniques and with calorimetry are analyzed. GuanidineHCl, urea, and SDS were studied as denaturants. The protein size ranged from 30 to ∼1600 amino acid residues, including a monoclonal antibody. The present model yields excellent simulations of all unfolding isotherms. The native protein is the reference state with a zero free energy. The free energy becomes negative upon unfolding and decreases with the logarithm of the denaturant concentration. The temperature dependence of the free energy provides the unfolding entropy and, in turn, the unfolding enthalpy. The latter agrees with the calorimetric measurements. The binding constant *K*_D_ depends essentially on the type of denaturant and varies little with the nature of the protein. The cooperativity parameter for chemical unfolding is compared to that for thermal unfolding.^11^ The multistate cooperative model is firmly grounded in statistical mechanical thermodynamics. With a multistate cooperative approximation, we provide a simple expression for cooperative chemical denaturation analysis, which is equally easy to apply as the LEM.

## Materials and Methods

Chemical unfolding experiments with guanidine HCl, urea, and SDS (subsequently performed with different spectroscopic and calorimetric techniques) are selected from the available literature. The focus is on the chemical unfolding of lysozyme with guanidineHCl, urea, and SDS. Altogether, 23 published chemical denaturation isotherms of proteins of different structure and size have been investigated.

### Chemical Unfolding Models

Chemical denaturants such as guanidineHCl and urea are commonly used to study protein stability. They change the polarity of the environment, bind to backbone and amino acid residues, and thus change the native protein conformation (N) into the unfolded conformation (U) (all-or-none folding). In contrast, the transition of individual amino acids from their native state (n) to their unfolded state (u) denotes multistate unfolding.

#### Multistate Cooperative Unfolding Model

The multistate cooperative model considers the individual amino acid residues of the protein. The amino acid residues in the native or in the unstructured confirmation are designated as “n″ and “u″. The initial step of this model is the binding of a denaturant D to an amino acid “n″ in a structured protein segment, inducing a conformational transition to an unstructured state "u":
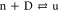
1

This chemical equilibrium is described by a simple equation:
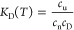
2

The concentrations *c*_n_ and *c*_u_ are the concentrations of the amino acid residues participating in unfolding. The binding constant *K*_D_(*T*) is a function of the temperature only. Unfolding is a dynamic equilibrium between many different protein conformations.

The statistical interpretation of a chemical equilibrium requires a grand canonical partition function. To make the connection to the textbook literature, we consider a system of only two types of particles. “In a grand canonical ensemble, the number of type A particles, *N*_A_, and type B particles, *N*_B_, are both variable. Let μ_A_ and μ_B_ be the respective chemical potentials of the two components. The grand partition function is
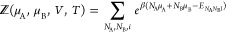
3(ref (12), chapter 13.9). Equation 2 defines a three-component system measuring the ensemble size in concentration units *c*_i_ (mol/L). The chemical potential is given by
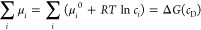
4
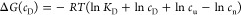
5

In chemical unfolding experiments, the protein concentration is typically ∼10 μM, whereas the concentration of the denaturant is 1–8 M. The chemical potential of the equilibrium is dominated by the denaturant concentration, leading to the following approximation.
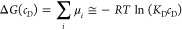
6

The conditional probability, *q*(*c*_D_) of a residue “n″ in a structured protein segment is defined as 1, the conditional probability of the unfolded residue “u″ is
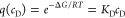
7provided the residue is located at the end of a native stretch of amino acid residues. If unfolding occurs in the middle of a native segment, unfolding is more difficult, and the corresponding conditional probability is σ*q*(*c*_D_) (σ ≪ 1). The conditional probability *q*(*c*_D_) is inserted into the Zimm–Bragg partition function *Z*(*c*_D_).^12^
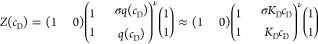
8

ν denotes the number of amino acid residues participating in the unfolding reaction. The cooperativity parameter σ determines the steepness of the unfolding transition. A small σ value leads to a sharp transition. The cooperativity parameter in chemical unfolding is typically σ ≈ 10^–2^–10^–3^ and is 10 to a hundred times larger than that of thermal unfolding_._ The fraction of unfolded protein is
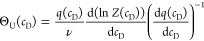
9

For a noncooperative ensemble with σ = 1, the partition function eq 8 reduces to *Z*(*q*) = (1 + *q*)^ν^. The fraction of unfolded protein becomes independent of ν and is,, which is identical to the Langmuir adsorption isotherm.

**Figure 1:**
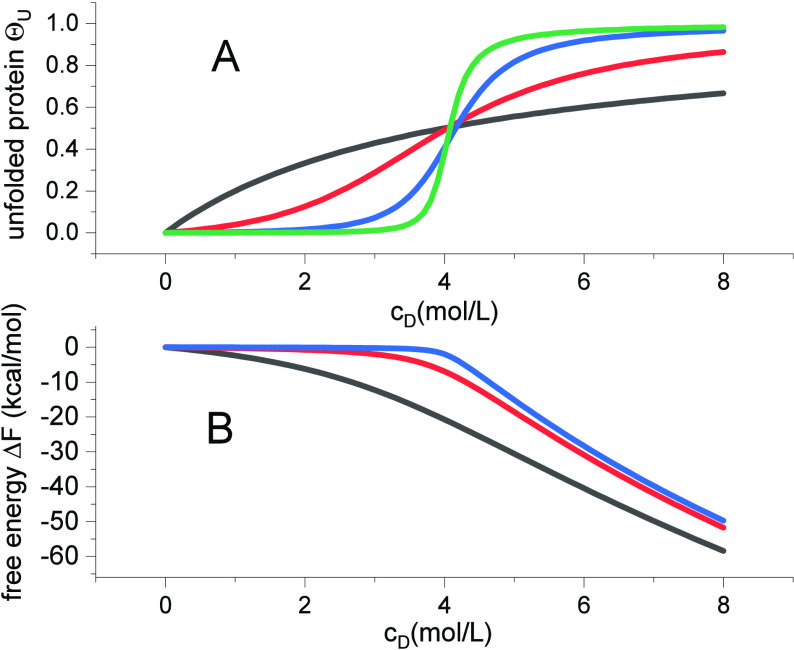
Multistate cooperative unfolding model. Variation of the cooperativity parameter σ. (A) Extent of unfolding Θ_U_(cD) (eq 9). Black: σ = 1. Red: σ = 10^–1^. Blue: σ = 10^–2^. Green: σ = 10^–3^ (B) Free energy as a function of cooperativity parameter σ and denaturant concentration (eq 10). Binding constant, *K*_D_ = 0.25 M^–1^. Midpoint concentration *c*_0_ = 4.0 M. Number of amino acid residues participating in transition *v* = 129. Temperature *T* = 298 K. The simulation parameters *K*_D_, *c*_0_, *v*, *T* and σ = 10^–3^ correspond to the unfolding of lysozyme in the guanidineHCl solution.

Figure 1 shows the unfolding isotherm Θ_U_(c_D_) (eq 9) for different cooperativity parameters σ. The binding constant is *K*_D_ = 0.25 M^–1^, which is typical for binding of guanidineHCl to most proteins. The binding constant is too small to induce complete protein unfolding for a noncooperative ensemble (σ = 1) as demonstrated by the Langmuir isotherm in Figure 1A. A dramatic change in the binding isotherm is induced by including even small cooperative interactions (red to green lines in Figure 1A).

The extent of unfolding (eq 9) is the result of the partition function *Z*(*c*_D_). Likewise, the free energy is also related to the partition function according to standard statistical thermodynamics.^13^ The free energy change of unfolding is
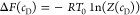
10Δ*F*(*c*_D_) upon addition of guanidineHCl is displayed in Figure 1B. The native protein is the reference state with zero free energy. Upon addition of denaturant, the free energy decreases. In the case of no cooperativity (σ = 1), the free energy decreases already at low concentrations of denaturant. For a cooperative ensemble, the free energy remains close to zero up to the midpoint of unfolding *c*_0_ and decreases rapidly beyond this concentration. Compared to noncooperative denaturation, the free energy change for a cooperative system is distinctly smaller.

The temperature dependence of the free energy is
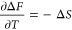
11

The unfolding enthalpy can then be calculated as
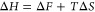
12

#### Simple Multistate Cooperative Approximation

For the biochemical practitioner, the above formalism may act as a deterrent. Fortunately, the matrix eq 8 can be replaced by a simple linear expression, which can easily be calculated. The cooperativity parameter in chemical unfolding experiments is always σ ≥ 10^–3^, and the largest eigenvalue λ_0_ of the above matrix is a sufficient approximation, resulting in a simpler partition function^12^
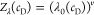
13with
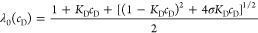
14

The fraction of unfolded protein is given by
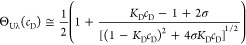
15

At the midpoint of unfolding with *c*_D_ = *c*_0_ and Θ(*c*_0_) = 1/2 follows
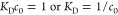
16

In the multistate cooperative model, the binding constant of the denaturant is simply the reciprocal of the midpoint concentration of unfolding. Equation 15 can also be written as
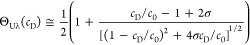
17

Equations 15 and 17 are equivalent to a cooperative sorption isotherm, which is based on a statistical–mechanical partition function. They are In line with many other sorption isotherms.^14^ The only fit parameter in the simulation of chemical unfolding isotherms is, therefore, the cooperativity parameter σ.

The free energy of the system is
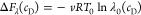
18

For denaturant concentrations *c*_*D*_ ≫ *c*_0_, the following approximation is valid
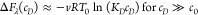
19

#### Concentration Gradient Δ*c* = *c*_end_ – *c*_ini_ and the Free Energy of the n → u transition

The multistate cooperative model predicts a one-to-one relationship between the free energy of the concentration gradient Δc = c_end_-c_ini_ and the energy required to induce the n → u transition. Unfolding takes place in the concentration interval *c*_ini_ ≤ *c*_D_ ≤ *c*_end_. The concentration gradient Δ*c* = *c*_end_ – *c*_ini_ constitutes a diffusive or osmotic free energy
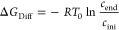
20

The free energy at ambient temperature is typically Δ*G*_Diff_ ≈ −0.4 to −1.0 kcal/mol. In parallel, the binding of D is associated with the free energy of the n → u transition per amino acid residue Δ*g*_nu_.
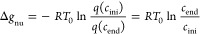
21

The predicted unfolding free energy per amino acid residue is thus identical to the concentration gradient (with opposite sign), that is, Δ*g*_nu_+Δ*G*_diff_ = 0. The multistate cooperative model is thus consistent with the thermodynamic expectation of a reversible equilibrium.

#### Chemical Equilibrium Two-State Unfolding

The common model to describe chemical unfolding isotherms is a noncooperative two-state model, which has dominated the field for the last 30–40 years. Only two types of protein conformations are assumed in solution, the native protein (*N*) and the unfolded protein (*U*). No intermediate structures and no specific interaction between denaturant D and protein are considered. The equilibrium N ⇄ U is simply
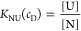
22

The equilibrium constant *K*(*c*_D_) varies with the concentration of denaturant. To calculate *K*_NU_(*c*_D_), the model makes an unconventional assumption about the free energy.
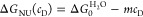
23

Equation 23 is difficult to understand in thermodynamic terms for two reasons. First, a large positive free energy Δ*G*_0_^H_2_O^ is assigned to the stable native protein. Second, the free energy is linear, not the usual logarithmic function of the concentration *c*_D_. The so-called linear extrapolation method allows the calculation of spectroscopic unfolding transitions. The fraction of unfolded protein Θ_U_(*c*_D_) is
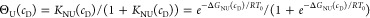
24

The disadvantage of this model is its conceptual simplicity of just two protein conformations and the rather questionable linear extrapolation method. It is generally believed that the native protein sits at a free energy minimum. In contrast, the LEM predicts a large positive free energy for a stable native protein.

By definition the midpoint concentration *c*_0_ is the concentration where native and unfolded protein occur at equal concentrations and the free energy is consequently zero in the LEM. It thus follows that
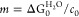
25*m* is not an independent variable, which is however often ignored in the analysis of spectroscopic data.

## Results

### Chemical Unfolding of Lysozyme with GuanidineHCl at 30 °C

**Figure 2:**
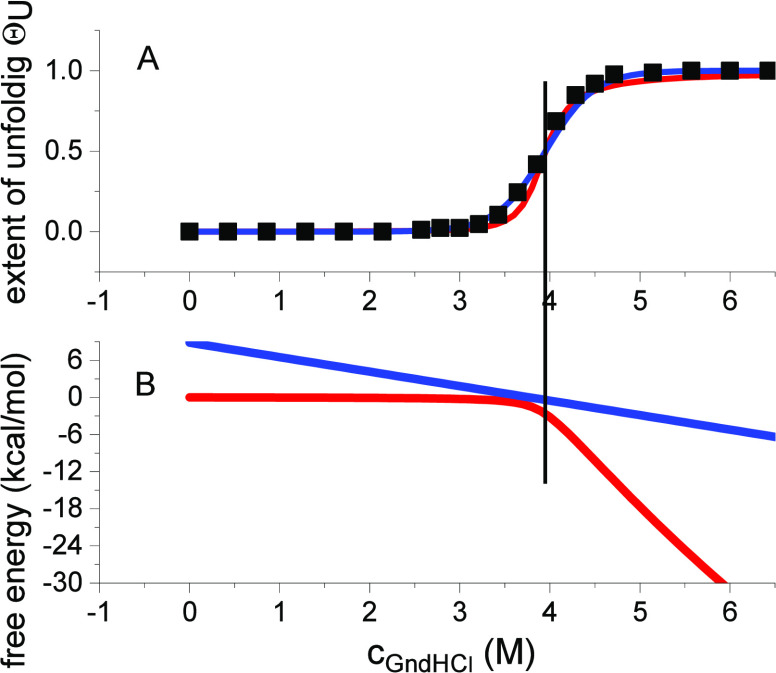
Unfolding of lysozyme in guanidineHCl solution at 30 °C. Red lines: multistate cooperative model. Blue lines: linear extrapolation method. Vertical lines: midpoint concentration *c*_0_ = 3.9 M. (A) (Box solid) data taken from ref (15). Simulation parameters of the multistate cooperative mode: *K*_D_ = 1/c_0_ = 0.26 M^–1^, σ = 1 × 10^–3^. LEM parameters: Δ*G*_0_^H_2_O^ = 8.837 kcal/mol, *m* = Δ*G*_0_^H_2_O^/*c*_0_ = 2.34 kcalL/mol^2^. (B) Temperature profiles of the free energy.

Figure 2 displays the chemical unfolding of lysozyme in guanidine HCl at 30 °C (experimental data from ref (15)). The midpoint concentration of unfolding is *c*_0_ = 3.9 M. Chemical unfolding takes place in the concentration range of 2.9 M ≤ *c*_D_ ≤ 5.3 M

The solid lines in Figure 2A simulate the experimental data with the parameters given in the legend of Figure 2. Both methods simulate the unfolding transitions equally well. Both models predict exactly 50% unfolding at the midpoint concentration *c*_0,_ but the concentration profiles of the free energy are quite different (Figure 2B). The LEM assigns a large positive free energy, Δ*G*_0_^H_2_O^ = 8.93 kcal/mol, to the native protein. At the midpoint concentration *c*_0_, the free energy is exactly zero (Δ*G*(*c*_0_) = 0). The multistate cooperative model predicts zero free energy for the native protein. The free energy change is slightly negative up to *c*_0_, (Δ*F* = −2.0 kcal/mol) and decreases rapidly at *c*_D_ > *c*_0_ (eq 18). The change in free energy upon unfolding (in the concentration range 2.9 M ≤ *c*_D_ ≤ 5.3 M is Δ*F* = −21.7 kcal/mol for the multistate cooperative model but only Δ*G* = −11.8 kcal/mol for the LEM.

### Lysozyme Unfolding in Urea

**Figure 3:**
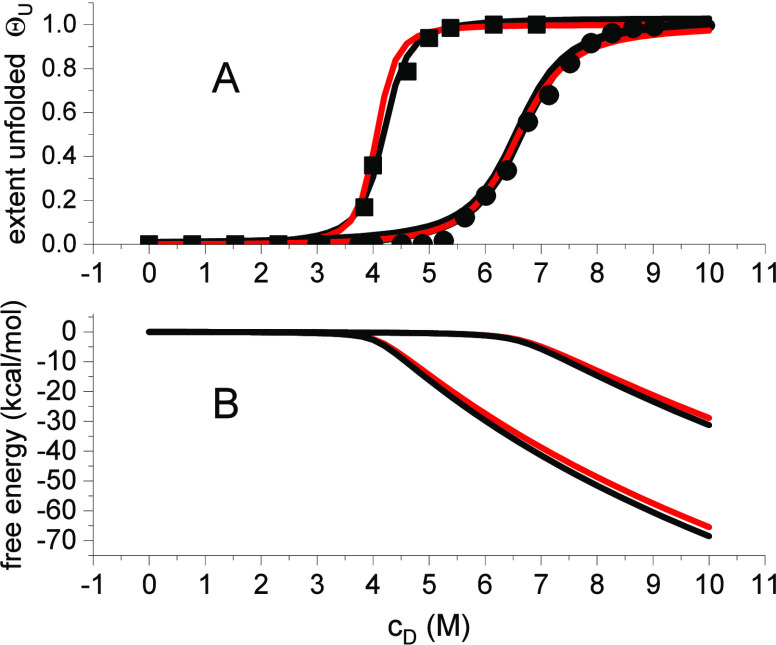
Chemical unfolding of lysozyme with guanidineHCl (box solid) and urea (circle solid) at 25 °C and pH 7. Data taken from ref (16). The midpoint concentrations *c*_0_ = 4.1 M for guanidine HCl cooperativity parameter is σ = 2 × 10^–3^ for both denaturants. The figure also compares the exact and approximate solutions for the binding isotherm and free energy. (A) Extent of unfolding. Red line: matrix solution eq 9. Black line: simplified isotherm eq 17. (B) Free energy of unfolding. Red line: eq 10. Black line: eq 18.

Urea is less effective in chemical denaturation than guanidineHCl as is obvious in Figure 3. The urea midpoint concentration is 6.7 M compared to 4.1 M for guanidine HCl. All thermodynamic data are summarized in Table 2, see ref (16).

The urea-induced transition (unfolding concentration range Δ*c* = 4.8 M) is broader than that of guanidineHCl (Δ*c* = 3 M). However, in spite of these large differences, the corresponding diffusive or osmotic free energies Δ*G*_diff_ (eq 20) constituted by these gradients are identical with Δ*G*_diff_ = −0.431 kcal/mol. Likewise, the free energy changes Δ*F* ≈ −24 kcal/mol and the cooperativity parameter σ = 2 × 10^–3^ are also identical for the two denaturants. The only significant thermodynamic difference is that the urea binding constant *K*_D_ = 0.15 M^–1^ is distinctly smaller than the guanidineHCl binding constant *K*_D_ = 0.245 M^–1^. The broadening of the transition region in the urea solution is thus caused by the low urea binding constant and not by a change in the protein cooperativity.

Figure 3 compares the exact and the approximate solutions for the extent of unfolding and (eqs 9 and 10 versus eqs 17 and 18). The approximate solutions show almost complete overlap with the exact solutions.

### Temperature-Dependence of Chemical Unfolding of Lysozyme

**Figure 4:**
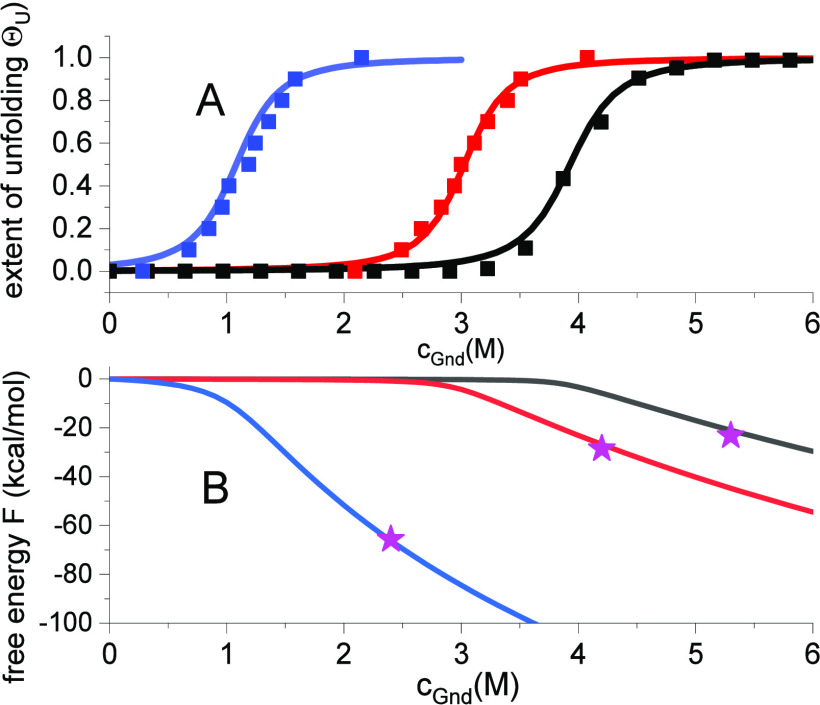
Temperature dependence of lysozyme unfolding in guanidineHCl solution. Data points taken from Figure 2 of ref (17). Solid lines are the simulations of the multistate cooperative model. Blue: 60 °C, K_D_ = 0.9 M^-1^, σ = 3 × 10^–2^. Red: 40 °C, K_D_ = 0.34 M^–1^, σ = 2.5 × 10^–3^. Black: 10 °C, K_D_ = 0.26 M^–1^, σ = 1 × 10^–3^. (A) Extent of unfolding Θ_U_(c_D_). Simulations with eq 9. (B) Free energy *F*(*c*_D_), calculated with eq 10. (pink star) Free energies at 95% unfolding, calculated with eq 19.

Lysozyme unfolding depends on the pH and temperature. A decrease in pH or an increase in temperature shift the unfolding transition toward lower c_0_ values. This is illustrated in Figure 4 for chemical denaturation with guanidineHCl at three different temperatures.

Figure 4A displays unfolding isotherms obtained with UV and CD spectroscopy.^17^ The solid lines are simulations with the multistate cooperative model. The binding constants *K*_D_ are exactly the reciprocal of the midpoint concentrations *c*_0._ The binding constants increase with temperature and the transition regions broaden. The cooperative interactions are reduced, leading to a larger cooperativity parameter σ. A temperature increase from 10 to 60 °C increases the cooperativity parameter by a factor of about 10.

Figure 4B displays the predicted concentration dependence of the free energy. The free energy is zero for the native protein and decreases rapidly beyond the midpoint of unfolding *c*_0_. The solid lines are calculated with the exact solution eq 9. The three stars-marked data points were calculated with eq 19, corresponding to 95% unfolding. The comparison with the exact solution demonstrates that the simple eq 19 is an excellent approximation for the free energy if *c*_d_ ≫ *c*_0_.

We have analyzed published denaturation experiments of lysozyme in guanidineHCl solutions at different temperatures, and the results are summarized in Table 1.

**Table 1:** Chemical Unfolding of Lysozyme with Guanidine HCl at Different Temperatures

temp	*c*_mid_	*K*_D_		*c*_ini_	*c*_end_	Δ*F*	*g*_den_		*m*
°C	M	M^–1^	σ	M	M	kcal/mol	kcal/mol	kcal/mol	kcal L/mol^2^
10^20^	3.8	0.263	1.50 × 10^–03^	2.7	5.3	–21.8	–0.379	9.55	2.512
15^20^	4.0	0.25	1.80 × 10^–03^	2.7	5.6	–22.7	–0.417	8.598	2.150
20^20^	3.9	0.257	2.00 × 10^–03^	2.6	5.5	–23.8	–0.436	8.598	2.210
25^20^	3.8	0.263	2.50 × 10^–03^	2.4	5.5	–26.1	–0.491	9.554	2.513
35^20^	3.3	0.303	5.00 × 10^–03^	1.75	5.1	–32.6	–0.654	8.598	2.605
10^17^	3.8	0.26	1.00 × 10^–03^	2.9	5.25	–20.1	–0.334	9.55	2.483
40^17^	2.9	0.34	2.50 × 10^–03^	1.9	4.1	–26.41	–0.493	8.36	2.842
60^17^	1.1	0.9	3.00 × 10^–02^	0.25	2.4	–65.5	–1.5	4.299	3.869
25^22^	3.9	0.256	1.00 × 10^–03^	2.8	5.15	–21.2	–0.361	10.03	2.568
10^21^	4.1	0.243	2.00 × 10^–03^	2.7	5.8	–22.55	–0.43	8.837	2.147391
30^15^	3.9	0.258	1.00 × 10^–03^	2.9	5.3	–21.7	–0.363	8.837	2.236
45^15^	3.2	0.31	1.00 × 10^–02^	1.3	5.3	–39.3	–0.888	4.777	2.236

Polypeptides and proteins of different sizes and structures were also analyzed. The fits of the experimental data with the multistate cooperative model were all of comparable quality as shown for lysozyme in Figures 2–4. The data are summarized in Table 2.

**Table 2:** Chemical Unfolding of Peptides and Proteins of Different Sizes and Structures in Guanidine HCl and Urea

protein, peptide	*N*_aa_	pH	denaturant	*K*_D_	*c*_mid_	σ	*c*_ini_	*c*_end_	Δ*c*	*g*_den_	Δ*F*		
				M^–1^	M		M	M	M	kcal/mol	kcal/mol	lit.	figure
BBL	41	7	GndHCl	0.33	3.03	3.00 × 10^–02^	0.2	7.5	7.3	–2.14	–20.9	(31)	Figure 2B
ubiquitin WT	76		GndHCl	0.34	2.94	7.00 × 10^–03^	1.4	5	3.6	–0.753	–22.1	(32)	Figure 1
ubiquitin L67S	76		GndHCl	0.72	1.39	1.30 × 10^–02^	0.5	2.7	2.2	–0.998	–28.4	(32)	Figure 1
ubiquitin	76	2	GnddHCl	0.395	2.53	8.00 × 10^–03^	1.2	4.5	3.3	–0.782	–24.1	(33)	Figure 4
ubiquitin	76	5.5	GndHCl	0.295	3.39	1.20 × 10^–02^	1.3	6.3	5	–0.934	–26.4	(33)	
Apo lipoprotein A1	120	8	GndHCl	1	1.00	2.00 × 10^–02^	0.3	2	1.7	–1.12	–49.7	(34)	Figure 1
Apo lipoprotein A1	122	7.4	GndHCl	0.952	1.05	5.00 × 10^–03^	0.6	1.7	1.1	–0.61	–32.8	(35)	Figure 7
Apo lipoprotein A1	122	7.4	GndHCl	0.95	1.05	3.00 × 10^–02^	0.3	2.3	2	–1.21	–55.9	(36)	
lysozyme	129	7	GndHCl	0.245	4.08	2.00 × 10^–03^	2.8	5.8	3	–0.431	–24.6	(16)	Figure 1
lysozyme	129	2	GndHCl	0.42	2.38	2.00 × 10^–02^	0.8	4.6	3.8	–1.03	–49.6	(37)	Figure 2A
lysozyme	129	2.5	GndHCl	0.48	2.08	5.00 × 10^–03^	1.1	3.2	2.1	–0.632	–31.1	(37)	Figure 1
PMS-Ct	145	4	GndHCl	0.52	1.92	1.00 × 10^–03^	1.1	2.5	1.4	–0.486	–22.7	(38)	Figure 4
PMS-Ct	241	4	GndHCl	0.513	1.95	4.00 × 10^–03^	1.1	2.7	1.6	–0.531	–45.3	(38)	Figure 4
average				0.551	2.22	0.012			2.931	-0.90			
STDV				0.256	0.93	0.0096			1.659	0.43			
Ac-tyr-(ala-glu-ala-ala-lys-ala)_5_-phe-NH_2_	32	7	urea	0.25	4.00	8.00 × 10^–02^						(6)	Figure 1
Ac-tyr-(ala-glu-ala-ala-lys-ala)_8_-phe-NH_2_	50	7.0	urea	0.2	5.00	1.00 × 10^–02^	2.2	9.8	7.6	–0.884	–18.1	(39)	Figure 1273 K
HPr protein	85	7	urea	0.215	4.65	8.00 × 10^–03^	2	8	6	–0.793	–26.2	(40)	Figure 1B288 K
cytochrome c	106	5	urea	0.2	5.00	4.00 × 10^–03^	2.9	7.7	4.8	–0.578	–25.1	(3)	
cytochrome c	106	7	urea	0.155	6.46	4.00 × 10^–03^	3.6	9.5	5.9	–0.574	–22.5	(3)	Figure 5
P22 1-domaiin	123	6	urea	0.3	3.33	9.00 × 10^–03^	1.5	5.6	4.1	–0.753	–35.18	(41)	Figure 2
lysozyme	129	2	urea	0.303	3.30	2.00 × 10^–02^	2.2	6.4	4.2	–0.632	–47.9	(37)	Figure 2B
lysozyme	129	5.5	urea	0.15	6.67	2.00 × 10^–03^	4.5	9.3	4.8	–0.43	–23.3	(16)	Figure 1
anti-EF-R mab	1600	7	urea	0.18	5.56	2.00 × 10^–02^	2	8.3	6.3	–0.85	–407	(3)	Figure 2
average				0.22	4.80	1.74 × 10^–02^			5.46	-0.69			
STDV				0.05	1.15	0.0229			1.12	0.15			
lysozyme	129		SDS	230.00	0.00	7.00 × 10^–02^	0.001	0.012	0.011	–1.47	–77.7	(18)	Table 1 298 K

### Chemical Unfolding with Sodium Dodecyl Sulfate (SDS)

**Figure 5:**
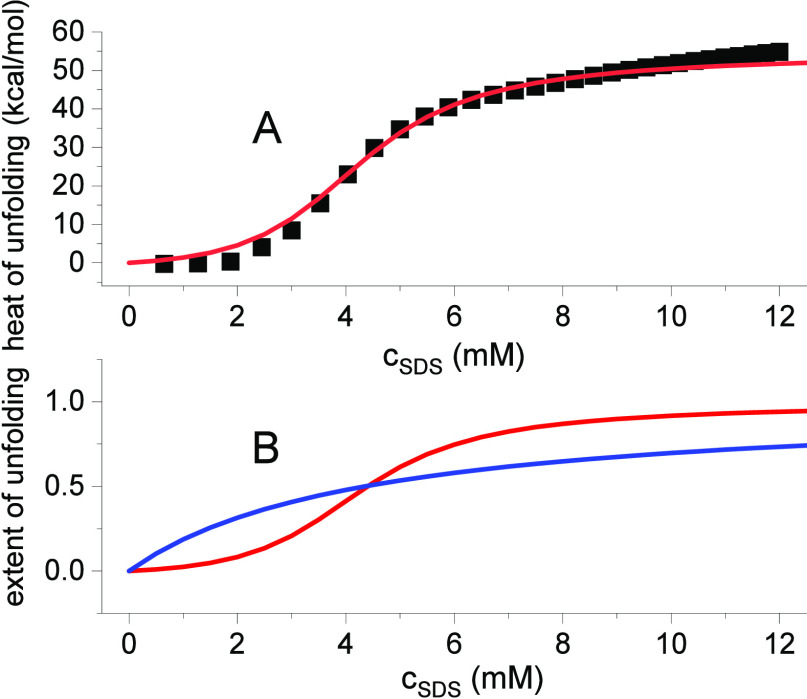
Calorimetric titration of 68 μM lysozyme with a SDS solution at 25 °C. (A) Experimental data taken from ref (18), Table 1. The published data were normalized to 1 mol lysozyme. Each data point is a separate measurement. Red line: multistate cooperative model. *K*_D_ = 230 M^–1^, σ = 7 × 10^–2^, enthalpy of unfolding Δ*H*_0_ = 55 kcal/mol. (B) Unfolding isotherm. Red line: multistate cooperative model, calculated with the same parameters as listed in panel (A). Blue line: *K*_D_ as in panel (A), but σ = 1 (noncooperative Langmuir isotherm).

SDS is a much stronger denaturant then guanidine HCl or urea as shown in Figure 5 (data taken from ref (18)). Unfolding was measured with calorimetry. At 25 °C, the midpoint concentration of unfolding is only 4.35 mM, resulting in a large binding constant of *K*_D_ = 230 M^–1^. The cooperativity of SDS-induced unfolding is relatively low with σ = 7 × 10^–2^.

The enthalpy of unfolding, Δ*H*_0_, is the most important parameter in the thermal unfolding studies. Spectroscopic measurements of chemical unfolding isotherms cannot provide this thermodynamic property. It is obtained, however, by a direct calorimetric measurement, as shown in Figure 5. Lysozyme is titrated with low concentrations of SDS, and the heat of reaction is measured in a calorimeter. Each data point in Figure 5 corresponds to an independent measurement.^18^ The total unfolding enthalpy is Δ*H*_n_ = 55 kcal/mol at 25 °C. The analogous measurement at 35 °C results in 61.3 kcal/mol, and the heat capacity change is Δ*C*_p_ = 0.65 kcal/molK. Likewise, the result of an early isothermal enthalpimetric titration of lysozyme with guanidineHCl at pH 2.5 and 40 °C yielded 56 kcal/mol.^19^

Altogether, 22 chemical unfolding isotherms of polypeptides and proteins of different size and structure were analyzed. The fits of the experimental data with the multistate cooperative model were all excellent. The results are summarized in Table 2.

## Discussion

### Characteristics of the Multistate Cooperative Model

The multistate cooperative model is based on a statistical–mechanical partition function that contains two molecular parameters: the binding constant *K*_D_ = 1/*c*_0_ and the protein cooperativity σ. The cooperativity parameter determines the steepness of the unfolding transition (Figure 1). The model takes into account the number ν of amino acid residues participating in the unfolding reaction and makes the following predictions. (i) The free energy of the n → u transition Δ*g*_nu_ is identical to the free energy provided by the concentration gradient Δ*G*_diff_ of complete unfolding (eqs 20 and 21). (ii) The free energy of the native protein is the reference state with a zero free energy. This is the minimum free energy of a stable protein. Unfolding requires energy, which is stored in the unfolded protein and can be delivered if the unfolded protein returns to its ground state. (iii) A simple approximation can be given (eqs 17–19), which fits the experimental data extremely well and is as easy to apply as the LEM.

### Temperature Dependence of Unfolding

Denaturant binding is a complex process. The denaturant binds to the protein backbone and to amino acid side chains. Binding can be electrostatic or hydrophobic. In addition, the denaturant can change the hydrophobicity of the hydration layer. The binding affinity will be different for different amino acid residues. The binding constant and the cooperativity parameter reported in this paper are average values of all possible interactions. An interpretation in terms of a specific binding model is not attempted.

**Figure 6:**
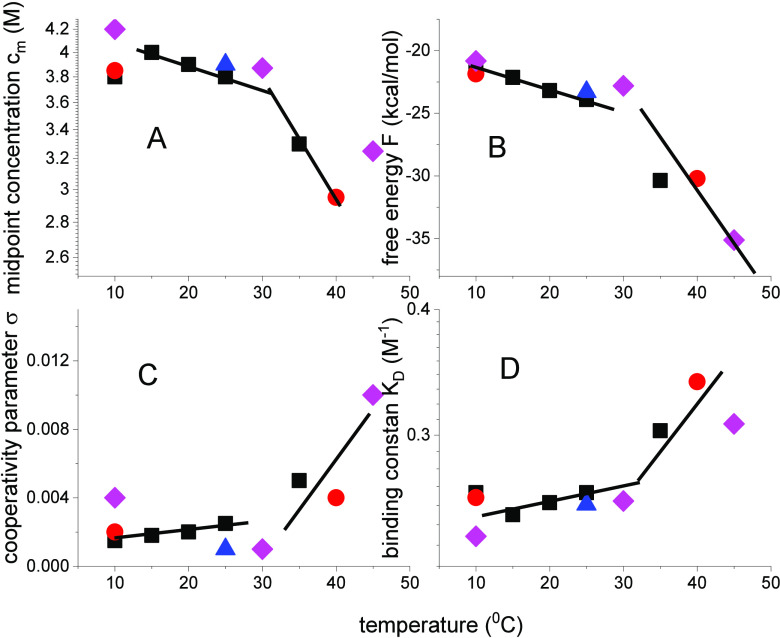
Lysozyme unfolding experiments in guanidineHCl at different temperatures and by different authors. (box solid),^20^ (circle solid),^17^ (pink diamond),^15,21^ (blue triangle)^22^. The solid lines are introduced to guide the eye.

The temperature dependence of the binding constant *K*_D_ and the cooperativity parameter σ together with that of the free energy is displayed in Figure 6 for lysozyme (Table 1). The simulation of the unfolding isotherms yields excellent fits in each case. Nevertheless, the scatter of the data in Figure 6 is considerable, as the isotherms are obtained by different authors under different experimental conditions. Two temperature regions can be discerned. Between 10 and 35 °C, the effect of temperature is small, above 35 °C all properties change rapidly.

The midpoint concentration and the free energy decrease with temperature, and the cooperativity parameter and the binding constant increase. At higher temperatures, the protein cooperativity decreases. The cooperativity parameter of chemical unfolding is at least 2 orders of magnitude larger than that of thermal unfolding.^11,23−25^ For example, the σ-parameter of lysozyme for chemical unfolding at 25 °C is σ = 1 × 10^–3^ (see Figure 2) but is only σ = 10^–6^ for temperature-induced unfolding (see Figure 1 in ref (11)). σ-Parameters of thermal unfolding of other proteins are found in refs (11,23−25). Chemical unfolding is less cooperative than thermal unfolding.

**Figure 7:**
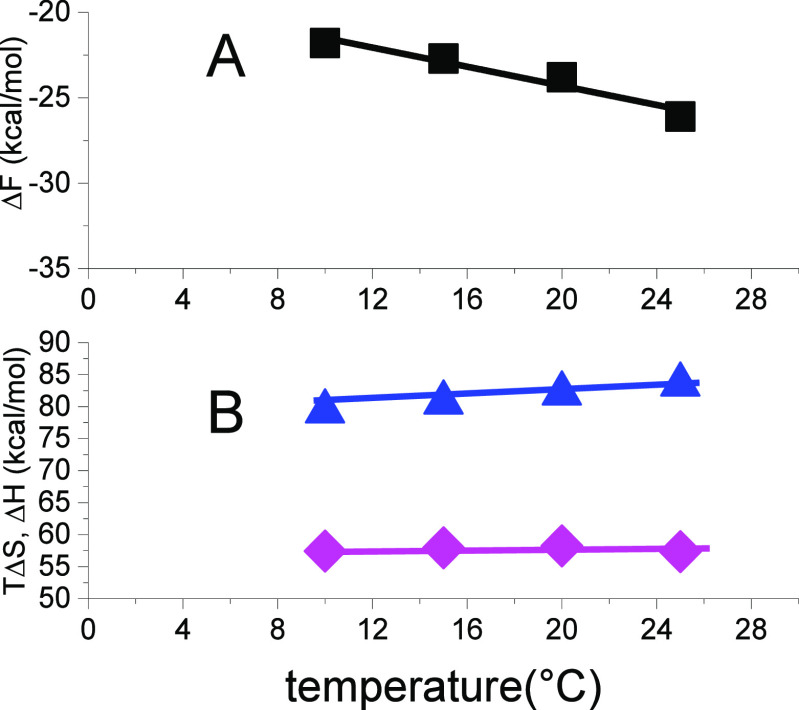
Chemical unfolding of lysozyme in guanidineHCl solution at different temperatures. (A) Free energy Δ*F*(*T*) calculated with the multistate cooperative model. Evaluation of unfolding isotherms of ref (20). Straight line: linear regression analysis with slope −Δ*S* = −0.28 ± 0.046 kcal/molK (R2 = 0.947). (B) (pink diamond) Enthalpy Δ*H* and (blue triangle) entropy *T*Δ*S*.

A detailed analysis of the temperature dependence of the free energy is given in Figure 7. Δ*H* = Δ*F* + *T*Δ*S*. Figure 7A displays data obtained from a consistent set of experiments for the temperature range 10 to 25 °C (Figure 1 in ref (20)).

From the negative slope of the straight line in Figure 7A, the entropy is calculated as Δ*S* = 0.280 ± 0.047 kcal/mol K. Figure 7B then displays the entropy term *T*ΔS and the predicted enthalpy Δ*H* = Δ*F* + *T*Δ*S*. The average enthalpy is Δ*H*_0_ = 58 ± 1 kcal/mol. An early isothermal titration study of lysozyme with guanidineHCl at pH 2.5 and 40 °C yielded Δ*H* = 56 kcal/mol.^26^ The present result is also in agreement with the SDS isothermal calorimetry shown in Figure 5, resulting in an enthalpy of 55 kcal/mol at 25 °C

The general understanding in the protein folding field has been that proteins fold into their native conformations driven by a decrease in free energy (negative Δ*G*).^7^ The native protein is thermodynamically the most stable conformation. In the so-called funnel hypothesis, the native protein sits at the bottom of a rough-edged funnel, representing the minimum free energy. The multistate cooperative model is fully consistent with this hypothesis. The native protein is the reference state with zero free energy, and unfolding requires energy. In returning reversibly to the native state, the unfolded protein loses its free energy.

### GuanidineHCl and Urea Binding Constants

Different molecular mechanism of denaturation have been proposed.^5,27^ An indirect mechanism postulates changes in the water structure and hydrophobic effect. The alternative view is the direct interaction of the chemical denaturant with the protein. Strong support for the latter mechanism comes from isothermal titration calorimetry (ITC) of guanidineHCl and urea with various proteins.^28^ X-ray studies also demonstrate a close interaction between guanidineHCl and the protein backbone of lysozyme.^29^

The guanidineHCl binding constants deduced from the lysozyme isotherms are 0.2 M^–1^≤ *K*_D_ ≤ 0.9 M^–1^ in the temperature range 10–60 °C (Table 1). Urea binds with a lower affinity of *K*_D_ = 0.15 M^–1^ (at 25 °C). Isothermal titration calorimetry was used to measure guanidineHCl and urea binding to different proteins.^28^ The data were analyzed by assuming a set of independent noncooperative binding sites.^28^ The binding constants deduced for lysozyme were *K*_D_ ≈ 0.4–0.8 M^–1^ for guanidine HCl and 0.06 M^–1^ for urea. This is in broad agreement with the results of the multistate cooperative model.

The binding constants of guanidineHCl and urea are small, and the binding of a single guanidineHCl molecule is not sufficient to induce the n → u transition of an amino acid residue. Only the cooperative binding of many denaturants induces protein unfolding.

The adsorption isotherm (eq 15) is also applicable to denaturation with organic solvents. Lysozyme denaturation isotherms have been reported for ethanol and DMSO.^30^ The midpoint concentrations and corresponding binding constants are 5 and 0.25 M^–1^ for ethanol and 7 and 0.17 M^–1^ for DMSO. In contrast, anionic SDS has a high affinity for overall cationic lysozyme.^18^ Based on Figure 1 of ref (18), the midpoint concentration is ∼4.3 mM and the binding constant *K*_D_ ≈ 230 M^–1^.
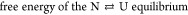


The multistate cooperative model provides a simple adsorption isotherm to analyze chemical unfolding (eq 15). The only unknown parameter is the cooperativity σ. The free energy follows from the partition function (eqs 10 or 18). The free energy of the native protein is zero, and the concentration profile is shown in Figure 1. Addition of denaturant initially leads to only a small negative free energy. The free energy becomes distinctly more negative at concentrations near and above the midpoint concentration *c*_0_. For large denaturant concentrations *c*_D_ ≫ *c*_0_, a simple approximation of Δ*F*(*c*_D_) ≈ −*RT*_0_*N*ln(*K*_D_*c*_D_) (eq 19) is valid. Numerical values of the free energy change Δ*F* = Δ*F*(*c*_end_) – Δ*F*(*c*_ini_), calculated with eq 10, are given in Table 1. The extent of unfolding in these calculations is 0.01 ≤ Θ_U_ ≤ 0.95. It should also be noted that the shape of the free energy in chemical unfolding (Figure 1) is identical to the free energy temperature profile in temperature-induced unfolding.^11^ However, the free energy change Δ*F* in chemical unfolding is about 3-fold higher more negative than the free energy of thermal unfolding.^11^ This must be traced back to the binding of the denaturants.

### Cooperativity in Chemical and Thermal Unfolding

Chemical σ parameters are plotted as a function of the temperature in Figure 6. Cooperativity parameter is σ ≃ 3 × 10^–3^ for temperatures below 30 °C and strongly increases at higher temperature. The increase in temperature facilitates chemical unfolding by decreasing the cooperativity of the protein. The free energy to start a new folded sequence within an unfolded region is given by Δ*F*_σ_ = −*RT*lnσ.

## Conclusions

It is obvious that the linear extrapolation method is a poor model to describe chemical unfolding. The two fit parameters have no well-defined thermodynamic or molecular basis. It is therefore attractive to modify an existing multistate cooperative model for thermal protein unfolding to address chemical unfolding. The new two-parameter model includes the binding constant of the denaturant and the cooperativity of protein unfolding. It is simple and easy to apply, as the binding constant is the reciprocal of the midpoint concentration of unfolding. Simple equations can be given for the binding isotherm and free energy. The free energy profile of chemical unfolding parallels the experimental results for thermal unfolding. The free energy of the native protein is zero and becomes distinctly negative upon unfolding only after reaching the midpoint concentration. The new model is satisfying as it provides an excellent description of chemical denaturation isotherms and for the first time allows comparing cooperative, chemical, and thermal unfolding.

## References

[ref1] Myers J. K.; Bell E.; Myers J. K. Chemical Denaturation. In Molecular Life Sciences; Springer: New York, NY; 2014.

[ref2] Grimsley G. R.; Trevino S. R.; Thurlkill R. L.; Scholtz J. M. Determining the conformational stability of a protein from urea and thermal unfolding curves. Curr. Protoc Protein Sci. 2013, 28, 28.4.1–28.4.1. 10.1002/0471140864.ps2804s71. 23377851

[ref3] Freire E.; Schon A.; Hutchins B. M.; Brown R. K. Chemical denaturation as a tool in the formulation optimization of biologics. Drug Discovery Today 2013, 18, 1007–1013. 10.1016/j.drudis.2013.06.005. 23796912 PMC3809824

[ref4] Pace C. N. Determination and analysis of urea and guanidine hydrochloride denaturation curves. Methods Enzymol. 1986, 131, 266–280. 10.1016/0076-6879(86)31045-0. 3773761

[ref5] Makhatadze G. I. Thermodynamics of protein interactions with urea and guanidinium hydrochloride. J. Phys. Chem. B 1999, 103, 4781–4785. 10.1021/jp990413q.

[ref6] Bolen D. W.; Yang M. Effects of guanidine hydrochloride on the proton inventory of proteins: implications on interpretations of protein stability. Biochemistry 2000, 39, 15208–15216. 10.1021/bi001071d. 11106500

[ref7] Sorokina I.; Mushegian A. R.; Koonin E. V. Is Protein Folding a Thermodynamically Unfavorable, Active, Energy-Dependent Process?. Int. J. Mol. Sci. 2022, 23, 521 10.3390/ijms23010521. 35008947 PMC8745595

[ref8] Zhou Y.; Hall C. K.; Karplus M. The calorimetric criterion for a two-state process revisited. Protein Sci. 1999, 8, 1064–1074. 10.1110/ps.8.5.1064. 10338017 PMC2144339

[ref9] Doig A. J. Recent advances in helix-coil theory. Biophys. Chem. 2002, 101–102, 281–293. 10.1016/S0301-4622(02)00170-9. 12488008

[ref10] Li-Blatter X.; Seelig J. Thermal and Chemical Unfolding of Lysozyme. Multistate Zimm-Bragg Theory Versus Two-State Model. J. Phys. Chem. B 2019, 123, 10181–10191. 10.1021/acs.jpcb.9b08816. 31686511

[ref11] Seelig J.; Seelig A. Molecular Understanding of Calorimetric Protein Unfolding Experiments. Biophys. Rep. 2022, 2, 100037 10.1016/j.bpr.2021.100037. PMC9680786 36425081

[ref12] Davidson N. Statistical Mechanics; Mac Graw-Hill: New York; 1962.

[ref13] Baumann R. P. Evaluation of thermodynamic properties In Modern Thermodynamics with Statistical Mechanics; McConnin R. A., Eds.; Macmillan Publishing Company: New York, USA; 1992; pp. 341.

[ref14] Adamson A. W. Physical chemistry of surfaces; John Wiley & Sons: New ork, N.Y., USA; 1976.

[ref15] Ibarra-Molero B.; Sanchez-Ruiz J. M. A model-independent, nonlinear extrapolation procedure for the characterization of protein folding energetics from solvent-denaturation data. Biochemistry 1996, 35, 14689–14702. 10.1021/bi961836a. 8942629

[ref16] Ahmad F.; Bigelow C. C. Estimation of the free energy of stabilization of ribonuclease A, lysozyme, alpha-lactalbumin, and myoglobin. J. Biol. Chem. 1982, 257, 12935–12938. 7130187

[ref17] Sasahara K.; Nitta K. Pressure-induced unfolding of lysozyme in aqueous guanidinium chloride solution. Protein Sci. 1999, 8, 1469–1474. 10.1110/ps.8.7.1469. 10422835 PMC2144367

[ref18] Behbeheni G. R.; Ramazani S.; Gonbadi K. A Thermodynamic Investigation on the Binding of Lysozyme with Sodium Dodecyl Sulfate. Chem. Soc. Pak. 2013, 35, 1427–1431.

[ref19] Pfeil W.; Privalov P. L. Thermodynamic investigations of proteins. II. Calorimetric study of lysozyme denaturation by guanidine hydrochloride. Biophys. Chem. 1976, 4, 33–40. 10.1016/0301-4622(76)80004-X. 1247649

[ref20] Sasahara K.; Sakurai M.; Nitta K. Pressure effect on denaturant-induced unfolding of hen egg white lysozyme. Proteins 2001, 44, 180–187. 10.1002/prot.1083. 11455591

[ref21] Laurents D. V.; Baldwin R. L. Characterization of the unfolding pathway of hen egg white lysozyme. Biochemistry 1997, 36, 1496–1504. 10.1021/bi962198z. 9063898

[ref22] Sasahara K.; Sakurai N.; Nitta K. The volume and compressibility changes of lysozyme associated with guanidinium chloride and pressure-assisted unfolding. J. Mol. Biol. 1999, 291, 693–701. 10.1006/jmbi.1999.2982. 10448047

[ref23] Seelig J.; Schönfeld H.-J. Thermal protein unfolding by differential scanning calorimetry and circular dichroism spectroscopy Two-state model versus sequential unfolding. Q. Rev. Biophys. 2016, 49, e9 10.1017/S0033583516000044. 27658613

[ref24] Seelig J.; Seelig A. Protein Stability. Analysis of Heat and Cold Denaturation without and with Unfolding Models. J. Phys. Chem. B 2023, 127, 3352–3363. 10.1021/acs.jpcb.3c00882. 37040567 PMC10123674

[ref25] Seelig J.; Seelig A. Protein Unfolding-Thermodynamic Perspectives and Unfolding Models. Int. J. Mol. Sci. 2023, 24, 5457 10.3390/ijms24065457. 36982534 PMC10049513

[ref26] Pace C. N.; Vajdos F.; Fee L.; Grimsley G.; Gray T. How to measure and predict the molar absorption coefficient of a protein. Protein Sci. 1995, 4, 2411–2423. 10.1002/pro.5560041120. 8563639 PMC2143013

[ref27] Konermann L. Protein unfolding and denaturants. In eLSJohn; Wiley & Sons, Ltd.: Chichester; 2012; 1–7.

[ref28] Makhatadze G. I.; Privalov P. L. Protein interactions with urea and guanidinium chloride. A calorimetric study. J. Mol. Biol. 1992, 226, 491–505. 10.1016/0022-2836(92)90963-K. 1322462

[ref29] Raskar T.; Koh C. Y.; Niebling S.; Kini R. M.; Hosur M. V. X-ray crystallographic analysis of time-dependent binding of guanidine hydrochloride to HEWL: First steps during protein unfolding. Int. J. Biol. Macromol. 2019, 122, 903–913. 10.1016/j.ijbiomac.2018.11.023. 30412756

[ref30] Sirotkin V. A.; Winter R. Volume Changes Associated with Guanidine Hydrochloride, Temperature, and Ethanol Induced Unfolding of Lysozyme. J. Phys. Chem. B 2010, 114, 16881–16886. 10.1021/jp105627w. 21117616

[ref31] Neuweiler H.; Sharpe T. D.; Johnson C. M.; Teufel D. P.; Ferguson N.; Fersht A. R. Downhill versus barrier-limited folding of BBL 2: mechanistic insights from kinetics of folding monitored by independent tryptophan probes. J. Mol. Biol. 2009, 387, 975–985. 10.1016/j.jmb.2008.12.056. 19136014

[ref32] Haririnia A.; Verma R.; Purohit N.; Twarog M. Z.; Deshaies R. J.; Bolon D.; Fushman D. Mutations in the hydrophobic core of ubiquitin differentially affect its recognition by receptor proteins. J. Mol. Biol. 2008, 375, 979–996. 10.1016/j.jmb.2007.11.016. 18054791 PMC2254529

[ref33] Ibarra-Molero B.; Loladze V. V.; Makhatadze G. I.; Sanchez-Ruiz J. M. Thermal versus guanidine-induced unfolding of ubiquitin. An analysis in terms of the contributions from charge-charge interactions to protein stability. Biochemistry 1999, 38, 8138–8149. 10.1021/bi9905819. 10387059

[ref34] Edelstein C.; Scanu A. M. Effect of guanidine hydrochloride on the hydrodynamic and thermodynamic properties of human apolipoprotein A-I in solution. J. Biol. Chem. 1980, 255, 5747–5754. 10.1016/S0021-9258(19)70693-0. 6769920

[ref35] Gwynne J.; Brewer B.; Edelhoch H. The molecular properties of ApoA-I from human high density lipoprotein. J. Biol. Chem. 1974, 249, 2411–2416. 10.1016/S0021-9258(19)42746-4. 4362678

[ref36] Mantulin W. W.; Pownall H. J. Reversible folding reactions of human apolipoprotein A-I: pressure and guanidinium chloride effects. Biochim. Biophys. Acta 1985, 836, 215–221. 10.1016/0005-2760(85)90069-4. 3927983

[ref37] Singh R.; Hassan M. I.; Islam A.; Ahmad F.; Khan R. H. Cooperative Unfolding of Residual Structure in Heat Denatured Proteins by Urea and Guanidinium Chloride. PloS One 2015, 10, e0128740 10.1371/journal.pone.0128740. 26046628 PMC4457810

[ref38] Santoro M. M.; Bolen D. W. Unfolding free energy changes determined by the linear extrapolation method. 1. Unfolding of phenylmethanesulfonyl alpha-chymotrypsin using different denaturants. Biochemistry 1988, 27, 8063–8068. 10.1021/bi00421a014. 3233195

[ref39] Scholtz J. M.; Barrick D.; York E. J.; Stewart J. M.; Baldwin R. L. Urea unfolding of peptide helices as a model for interpreting protein unfolding. Proc. Natl. Acad. Sci. 1995, 92, 185–189. 10.1073/pnas.92.1.185. 7816813 PMC42842

[ref40] Nicholson E. M.; Scholtz J. M. Conformational stability of the Escherichia coli HPr protein: test of the linear extrapolation method and a thermodynamic characterization of cold denaturation. Biochemistry 1996, 35, 11369–11378. 10.1021/bi960863y. 8784192

[ref41] Newcomer R. L.; Fraser L. C. R.; Teschke C. M.; Alexandrescu A. T. Mechanism of Protein Denaturation: Partial Unfolding of the P22 Coat Protein I-Domain by Urea Binding. Biophys. J. 2015, 109, 2666–2677. 10.1016/j.bpj.2015.11.010. 26682823 PMC4699920

